# *Elizabethkingia* bloodstream infections in severely immunocompromised patients: persistent, relapsing and associated with high mortality

**DOI:** 10.1093/jacamr/dlae161

**Published:** 2024-10-25

**Authors:** Mark Fahmy, Adam Stewart, Siok-Keen Tey, Krispin Hajkowicz

**Affiliations:** Pathology Queensland, Microbiology, James Mayne Building, Royal Brisbane and Women’s Hospital, Butterfield Street, Brisbane, Queensland 4006, Australia; Faculty of Medicine, Mayne Medical School, University of Queensland, 20 Weightman Street, Herston, Queensland 4006, Australia; Infectious Diseases, Sunshine Coast University Hospital, 6 Doherty Street, Birtinya, Queensland 4575, Australia; Faculty of Medicine, Mayne Medical School, University of Queensland, 20 Weightman Street, Herston, Queensland 4006, Australia; Haematology, Royal Brisbane and Women’s Hospital, Butterfield Street, Herston, Queensland 4006, Australia; Infectious Diseases, Royal Brisbane and Women’s Hospital, Butterfield Street, Herston, Queensland 4006, Australia; Infectious Diseases, University of Queensland Centre for Clinical Research, Building 71/918 RBWH Herston, Brisbane, Queensland 4006, Australia

## Abstract

**Objectives:**

*Elizabethkingia* species are uncommon causes of bloodstream infections, representing a true opportunistic and multi-drug-resistant pathogen to immunocompromised or vulnerable hosts. Despite this, data are lacking regarding optimal management strategy for infections with this organism, which is associated with significant mortality and morbidity. We describe patient characteristics, management and outcomes in this case series.

**Patients and methods:**

All inpatients at the Royal Brisbane and Women’s Hospital with a positive blood culture for *Elizabethkingia* spp. were identified by database query. Clinical information including medical history, source of infection, attempts at source control and outcome were collected. Laboratory data including duration of bacteraemia and antimicrobial susceptibility testing were also collected.

**Results:**

All patients had severe defects of innate and adaptive immunity. Targeted therapy was started promptly and efforts at source identification and control were appropriately pursued. Despite this, outcomes were generally poor. A previously unrecognized presentation of relapsing infection was described in one case, requiring long-term suppressive antimicrobials to control. One case died as a result of infection and one case was cured, but died soon after due to complications of immunosuppression.

**Conclusions:**

Treatment of these organisms is challenging due to limited effective therapy, development of on treatment resistance and profound host immunocompromise. Up-front use of multiple, optimally dosed antimicrobials, attempting source control and attempting to restore host immune function all appear to be key to achieving good outcomes.

## Introduction

Bacteria in the genus *Elizabethkingia* (*anophelis*, *endophytica*, *meningoseptica* and *miricola*) were identified as human pathogens by Dr Elizabeth King in 1959.^1^ These organisms are found globally in in soil, water and hospital environments.^2^ Severe infections are described in immunocompromised or vulnerable patients, such as patients undergoing chemotherapy, transplantation^3^ or premature infants.^4^*Elizabethkingia* spp. also pose risks for community and healthcare-associated outbreaks.^5^

*Elizabethkingia* spp. are impervious to chlorination and other disinfectants^6^ and are intrinsically resistant to carbapenems, aminoglycosides and most beta-lactams with quinolones often used for treatment.^7^ Resistance emerges on treatment, which limits antimicrobial options and can lead to treatment failure and death.^8^ Clinical data are lacking on antimicrobial regimens for adult patients with severe *Elizabethkingia* species infection. These patients are often multi-morbid, severely immunocompromised and have poor bacterial clearance.^9^

We highlight three cases from the same centre of persistent, refractory and relapsing *Elizabethkingia* spp. bloodstream infections in severely immunocompromised patients, focusing on treatment, antimicrobial resistance and clinical outcomes.

## Patients and methods

### Case 1

**Figure 1.**
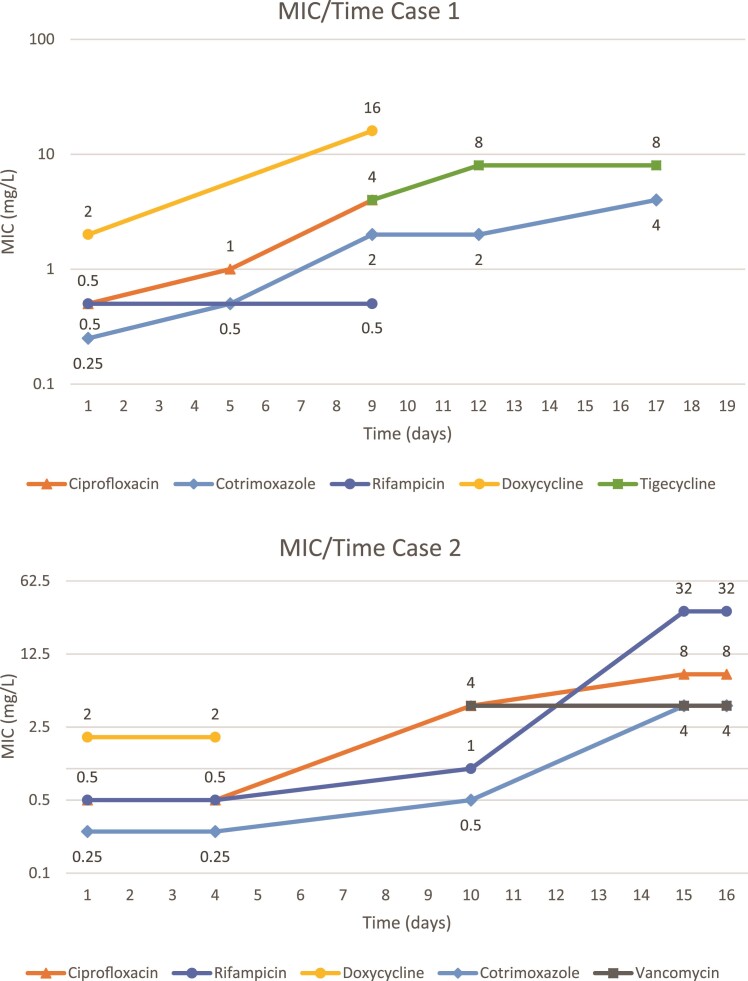
Graphs demonstrating MIC over time for Cases 1 and 2.

A 41-year-old man with a background of allogenic stem cell transplantation for chronic myeloid leukaemia-myeloid blast crisis 8 years previously presented with fever and left leg cellulitis. He was taking prednisolone 25 mg daily and ruxolitinib 5 mg twice daily for severe chronic graft versus host disease (GVHD) affecting his skin and gastrointestinal tract. He recalled minor abrasions from cleaning his swimming pool. Eight years previously, hypotension associated with a dose of piperacillin-tazobactam had been labelled as anaphylaxis. He started empiric antibiotics with meropenem 1 g three times a day. Aerobic blood culture (BACT/ALERT^®^ Virtuo^®^) was positive for motile Gram-negative bacilli. Oxidase positive colonies grew on 5% horse blood agar (HBA) and MALDI–TOF MS (VITEK MS^®^ v.3.2; bioMerieux) identified *Elizabethkingia anophelis*. He was changed to ciprofloxacin 600 mg intravenously three times daily. He remained bacteraemic and was commenced on cotrimoxazole 320/1600 mg PO twice daily on day 7, doxycycline 100 mg PO twice daily on day 8 and tigecycline intravenously 50 mg twice daily on day 15. Owing to ongoing bacteraemia and emerging resistance to ciprofloxacin and doxycycline (Figure 1) the historical reaction to piperacillin-tazobactam was reconsidered. This event occurred during a blood transfusion without signs of anaphylaxis. He had no adverse reactions to a test dose of piperacillin-tazobactam allowing multi-drug therapy on day 20 with cotrimoxazole 320/1600 mg PO twice daily, piperacillin-tazobactam 4.5 g four times a day and minocycline intravenously 100 mg twice a day. Blood cultures became negative on day 20 and treatment continued until day 34 with resolution of his cellulitis. Investigations for a persistent focus including whole body positron-emission tomography/computer tomography, left leg ultrasound and trans-oesophageal echocardiogram were unrevealing.

The patient returned on day 44 with recurrent left leg cellulitis, fever and shock requiring ICU admission for vasopressor support. Empiric therapy for suspected necrotizing fasciitis with clindamycin and piperacillin-tazobactam was started. Later that day, therapy was changed to piperacillin-tazobactam 4.5 g four times a day, minocycline intravenously 100 mg twice daily and cotrimoxazole 320/1600 mg PO twice daily when blood cultures grew *Elizabethkingia anophelis*. There had been no further environmental exposures. Investigations for persistent infection were unrevealing apart from a cavitary lung lesion secondary to invasive aspergillosis, representing a high total burden of immunosuppression. He completed 2 weeks of therapy before changing to cotrimoxazole 160/800 mg PO twice daily and minocycline 100 mg PO twice daily for long-term suppression.

One hundred and 91 days after his initial presentation, a second relapse occurred. He presented with fatigue, malaise and fevers with no clear source of infection. Blood cultures again grew *Elizabethkingia anophelis* and he commenced therapy with piperacillin-tazobactam 4.5 g four times a day with cotrimoxazole 160/800 mg PO twice daily and minocycline PO 100 mg twice daily. His steroids were reduced to prednisolone 17.5 mg daily (from 25 mg daily). He continued intravenous therapy for 8 days, before continuing cotrimoxazole and minocycline as suppressive therapy.

### Case 2

A 72-year-old man with a background of Waldenstrom’s macroglobulinaemia, type 2 diabetes mellitus, hypertension, asthma and gastroesophageal reflux disease presented with fever, headaches, otalgia and meningism. He had been treated with bendamustine and rituximab for Waldenstrom’s 10 months previously. He was diagnosed with meningitis with otomastoiditis requiring ICU admission for shock and delirium. He commenced ceftriaxone 2 g twice a day and then meropenem 2 g three times a day. Blood and CSF cultures both grew oxidase positive, Gram-negative bacilli on HBA that MALDI–TOF MS (VITEK MS^®^ v 3.2; bioMerieux) identified as *Elizabethkingia meningioseptica*. Antibiotics were changed to ciprofloxacin 400 mg intravenously three times a day, increased to 600 mg three times a day on day 14 due to an increase in MIC (Figure 1). He underwent mastoidectomy and grommet insertion on day 4 for source control. Owing to persistent bacteraemia cotrimoxazole 320/1600 mg intravenously three times a day was added to his therapy, which was discontinued 5 days later due to presumed drug-induced liver injury. At this stage rifampicin 300 mg PO twice daily and piperacillin-tazobactam 24.5 g/24 hours continuous intravenous infusion were initiated. Owing to continued deterioration and bacteraemia intra-ventricular vancomycin 20 mg daily was attempted for 3 days. Unfortunately, despite treatment he died at day 23. He was bacteraemic for at least 17 days, although blood cultures were not performed on his last 5 days of life due to perceived futility.

### Case 3

A 61-year-old man was admitted to hospital for bone marrow transplantation for blastic plasmacytoid dendritic cell neoplasm. His recovery was complicated by neutropenic septic shock (neutrophil count 0.01 × 10^9^/L) requiring ICU admission 12 days post-transplant. He commenced piperacillin-tazobactam 4.5 g four times a day and then meropenem 1 g three times a day empirically. Blood cultures were positive for motile, oxidase positive Gram-negative bacilli, which were identified by MALDI–TOF MS (VITEK MS^®^ v.3.2; bioMerieux) as *Elizabethkingia anophelis.* He was changed to ciprofloxacin 400 mg intravenously three times a day on day 2. A Hickmann line presumed to be the source of infection was removed on day 3 and he received a single dose of tigecycline intravenously 200 mg. He continued to be bacteraemic after line removal, although he retained a newly inserted central venous line for drug administration. Cotrimoxazole 160/800 mg PO twice a day was added on day 7, with no further positive blood cultures. His neutrophil count increased to 0.29 × 10^9^/L on day eight, and to 1.31 × 10^9^/L on day nine. Antibiotic therapy was discontinued on day 10. Unfortunately, he died at day 51 due to complications of steroid refractory GVHD.

## Discussion

All cases had severe, persistent defects in innate and adaptive immunity. Only one case had significant neutropenia with count recovery appearing to contribute to blood culture clearance. This implies that while neutrophil function is an important part of host immune response, other factors are also necessary for good outcomes. In significant infections, investigation for immune defects and their correction may contribute to better outcomes.

Case 1 highlights a potentially unrecognized presentation of recurrent *Elizabethkingia* spp. infection. It is notable that this case appears to have been acquired cutaneously from water/environmental inoculum and relapses do not appear secondary to uncontrolled source. We speculate that the patient’s gastrointestinal tract may be colonized with *Elizabethkingia anophelis* with flares of GVHD allowing for bacterial translocation. Further metagenomic characterization of the patient’s faecal microbiome is underway to identify the source of infection.

Resistance to antibiotics used for Gram-negative infections (cephalosporins, carbapenems and aminoglycosides) was common.^7^ While later generation cephalosporins appear ineffective, combinations of beta-lactam/beta-lactamase inhibitors appear to have *in vitro* activity (Table 1) and probably *in vivo* effectiveness as seen with piperacillin-tazobactam in case 1, making them potentially useful agents when treating these organisms.

**Table 1.** Summary of patient characteristics, outcomes and laboratory investigations

**Case**	1	2	3
**Age**	41	72	61
**Sex**	Male	Male	Male
**Date of infection (year)**	2023	2021	2020
**Clinical factors**	Allogeneic stem cell transplant, Chronic GVHD, Functional asplenia, Active immunosuppression with prednisone (25 mg) and ruxolitinib (JAK21/inhibitor), Type 2 diabetes mellitus	Recent chemotherapy (bendamustine and rituximab), Lymphopenia (CD4^+^ T cells 50/µL, CD8^+^ T cells 30/µL, B cells <10/µL), Type 2 diabetes mellitus	Allogeneic stem cell transplant, Recent chemotherapy (cytarabine, idarubicin) and venetoclax (Bcl-2 inhibitor), Active immunosuppression with ciclosporin and methotrexate, Lymphopenia (CD4^+^ T cells 150/µL CD8^+^ T cells 460/µL B cells <10/µL), and neutropenia (<0.01 × 10^9^/L)
**Suspected Source of infection (initial)**	Left leg cellulitis	Meningitis with otomastoiditis	Hickman line associated
**Source control attempted**	n/a	Mastoidectomy with left grommet insertion	Line removed
**Time to source control from 1st positive culture (days)**	n/a	1	3
**Time to 1st positive blood culture (hours)**	14.6	17	15.5
**Time from positive culture to appropriate therapy (days)^a^**	2	1	1
**Duration of bacteraemia (days)**	20	17	7
**Outcome**	Relapsed infection	Death	Cure (non-infection-related death)
**Antimicrobial susceptibility testing^b^**	**Initial MIC (mg/mL)/disc zone (mm) (interpretation if available: CLSI non-*Enterobacterales* breakpoints M100-ED34:2024 used)**		
Amikacin	—	128 (R)	—
Azithromycin	>256 (R)	—	—
Aztreonam	6 mm (R)	—	—
Cefepime	—	64 (R)	—
Cefiderocol	16 mm (NC)	—	—
Ceftazidime	—	>256 (R)	—
Ceftazidime-avibactam	—	—	12 (NC)
Ceftolozane-tazobactam	—	—	4 (NC)
Ciprofloxacin	0.5 (S)	0.5 (S)	0.5 (S)
Co-trimoxazole	0.25 (S)	0.25 (S)	0.5 (S)
Doxycycline	2 (S)	2 (S)	—
Imipenem	32 (R)	—	—
Meropenem	—	—	>32 (R)
Minocycline	23 mm (S)^c^	—	—
Piperacillin-tazobactam	8 (S)	8 (S)	8 (S)
Rifampicin	0.5 (NC)	0.5 (NC)	0.5 (NC)
Tigecycline	4 (NC)	8 (NC)	4 (NC)
Vancomycin	16 (NC)	4 (NC)	—

MIC, minimum inhibitory concentration (mg/L), S, sensitive; R, resistant; NC, not calibrated; CLSI, Clinical and Laboratory Standards Institute.

^a^Appropriate therapy was defined as at least one agent with a susceptible result from available testing.

^b^Susceptibility testing with interpretation (S, I, R) performed by E-test methodology on Mueller–Hinton agar. Where disc diffusion was performed or breakpoints lacking, NC is used.

^c^Inferred from doxycycline.

In all cases, ciprofloxacin was used early in treatment. It is notable that MICs rose for all agents as demonstrated in Figure 1. Development of resistance was faster for ciprofloxacin than other agents due to the lower barrier for resistance to fluoroquinolones in Gram-negative organisms,^10^ precluding use as monotherapy in complicated infections with high bacterial burden. This may limit treatment options to sulphonamides and tetracyclines for difficult to treat infections.

*Elizabethkingia* spp. are susceptible to agents atypical for Gram-negative organisms including vancomycin and rifampicin, which have been trialled in severe infections. In case 2, vancomycin was unhelpful, with elevated MICs noted in Figure 1. Existing data suggest higher rates of clinical failure with vancomycin.^11^ Additionally, the *VanW* gene commonly expressed in *Elizabethkingia* spp. isolates from Australia^8^ is also found in microorganisms with VanB-type glycopeptide resistance.^12^ Usefulness of rifampicin is uncertain without evidence to recommend it for Gram-negative infections.^13^ There may be issues with toxicity or drug–drug interactions, as in case 1 where rifampicin was contraindicated due to an interaction with ruxolitinib.

Because these organisms are rarely isolated, high-quality evidence is difficult to produce, perhaps requiring development of centres for expertise in difficult to manage infections in immunocompromised hosts. This could take the form of referral centres with integrated antibiotic susceptibility testing and genomic capabilities, as well as collating databases of antimicrobial therapy and patient outcomes to guide recommendations.

It appears that key elements of management include early suspicion of *Elizabethkingia* spp. infection in patients with Gram-negative bacteraemia not responding to standard therapy, early efforts at source control where possible, attempting to reduce immunosuppression or augment host immune response if possible, up-front use of multiple agents, including beta-lactam beta-lactamase inhibitor combinations with optimized dosing and caution around use of fluoroquinolone monotherapy to avoid development of on treatment resistance.
